# Linkage Analysis and Multi-Locus Genome-Wide Association Studies Identify QTNs Controlling Soybean Plant Height

**DOI:** 10.3389/fpls.2020.00009

**Published:** 2020-02-14

**Authors:** Yanlong Fang, Shulin Liu, Quanzhong Dong, Kaixin Zhang, Zhixi Tian, Xiyu Li, Wenbin Li, Zhongying Qi, Yue Wang, Xiaocui Tian, Jie Song, Jiajing Wang, Chang Yang, Sitong Jiang, Wen-Xia Li, Hailong Ning

**Affiliations:** ^1^ Key Laboratory of Soybean Biology, Ministry of Education/Key Laboratory of Soybean Biology and Breeding/Genetics, Ministry of Agriculture, Northeast Agricultural University, Harbin, China; ^2^ State Key Laboratory of Plant Cell and Chromosome Engineering, Institute of Genetics and Developmental Biology, Chinese Academy of Sciences, Beijing, China

**Keywords:** soybean, plant height, quantitative trait nucleotides, multi-locus genome-wide association studies, linkage analysis

## Abstract

Plant height is an important target for soybean breeding. It is a typical quantitative trait controlled by multiple genes and is susceptible to environmental influences. Here, we carried out phenotypic analysis of 156 recombinant inbred lines derived from “Dongnong L13” and “Henong 60” in nine environments at four locations over 6 years using interval mapping and inclusive composite interval mapping methods. We performed quantitative trait locus (QTL) analysis by applying pre-built simple-sequence repeat maps. We detected 48 QTLs, including nine significant QTLs detected by multiple methods and in multiple environments. Meanwhile, genotyping of all lines using the SoySNP660k BeadChip produced 54,836 non-redundant single-nucleotide polymorphism (SNP) genotypes. We used five multi-locus genome-wide association analysis methods to locate 10 quantitative trait nucleotides (QTNs), four of which overlap with previously located QTLs. Five candidate genes related to plant height are predicted to lie within 200 kb of these four QTNs. We identified 19 homologous genes in Arabidopsis, two of which may be associated with plant height. These findings further our understanding of the multi-gene regulatory network and genetic determinants of soybean plant height, which will be important for breeding high-yielding soybean.

## Introduction

Soybean [Glycine max (L.) Merr.] is an important crop around the world, and worldwide soybean consumption has increased rapidly in recent years. Increasing production per unit area thus remains an important breeding target. Yield is influenced by a variety of traits, such as number of pods, number of seeds, and number of nodes. Plant height is an important factor in the formation of yield traits, and at the same time promotes or inhibits other yield components. Plant height mainly affects yield by influencing lodging resistance and the number of pods per plant (Akhter and Sneller, 1996). With the development of molecular genetics and molecular marker technology (Keim et al., 1990), the traditional breeding process has been greatly improved, producing cultivars with excellent traits.

Most agronomic traits in soybean, such as plant height and pod number, are controlled by quantitative trait loci (QTLs) (Mansur et al., 1996), making it difficult to understand their genetic basis and molecular mechanism due to insufficient precision. A large number of plant height-related QTLs have been reported. Specht et al. (2001) used a recombinant inbred line (RIL) population containing 236 individuals derived from a cross between “Minsoy” and “Noir 1” to identify nine soybean plant height QTLs distributed on eight chromosomes. Sun et al. (2006) used a RIL population containing 143 individuals derived from a cross between “Charleston” and “Dongnong 59” to uncover 17 plant height QTLs distributed on 10 chromosomes. Guzman et al. (2007) identified three soybean plant height QTLs using three backcross-derived populations. Yao et al. (2015a) used an F2-derivative group constructed from “Jiyu50” and “Jnong18” and containing 236 individuals to identify nine plant height QTLs distributed across linkage groups A1, C2, G, M, F, and K.

Genome-wide association studies (GWAS) exploit the abundant mutations and recombinations existing in diverse populations. They have a relatively high localization accuracy compared to traditional QTL mapping methods, enabling prediction and identification of genes. With the development of genotyping and sequencing technologies, GWAS have been widely used to study the genetic basis of traits in major crops such as maize (Poland et al., 2011; Li et al., 2013; Feng et al., 2015), rice (Huang et al., 2010; Bandillo et al., 2013), wheat (Huang et al., 2012; Sukumaran et al., 2015), *Arabidopsis thaliana* (Kover et al., 2009), and tomato (Zhang et al., 2016). GWAS are also widely used for genetic analysis of major traits in soybean such as flowering time (Zhang et al., 2015b), pod number (Fang et al., 2017), seed oil (Zhou et al., 2015), seed protein (Bandillo et al., 2015), salt tolerance (Kan et al., 2015), and 100-seed weight (Zhang et al., 2015a). However, there are few reports on GWAS of high-density single-nucleotide polymorphisms (SNPs) in soybean (Hwang et al., 2014). Therefore, the application of GWAS in soybean, especially for agronomic traits, remains to be explored. Previous studies have shown that soybean plant height is a typical quantitative trait controlled by multiple genes, and its genetic effect is relatively small. However, more effective methods are required for detecting QTLs. Multi-locus GWAS is a suitable method for detecting significant quantitative trait nucleotides (QTNs) and has been used in several studies (Hou et al., 2018; Zhang et al., 2018c).

In addition to natural populations, GWAS are widely used for genetic analysis of other mapping populations, such as nested association mapping (NAM) and multi-parent advanced generation inter-cross (MAGIC) populations (Korte and Farlow, 2013). Tian et al. (2011) used a maize NAM population for GWAS to determine the genetic basis of important leaf structural features. Cook et al. (2012) constructed a NAM population of 25 RILs and identified beneficial alleles for improving maize kernel starch, protein, and oil. Zhang et al. (2018a) used a four-way RIL to perform GWAS on the genetic mechanism of soybean protein. Henning et al. (2016) conducted GWAS using a hop population constructed from the parents “Teamaker” and USDA 21422M, revealing the genetic basis of fiber quality traits and yield components in 231 lines. Liu et al. (2018) used a F_6:8_ RIL derived from cotton cultivars “Lumianyan28” and “Xinluzao24” to detect 134 QTLs for fiber quality traits and 122 QTLs for yield components, with 35 common candidate genes. Therefore, analyzing the genetic basis of complex traits in single bi-parental populations is feasible using GWAS. At the same time, Ott et al. (2011) consider that linkage analysis and association analysis together are more accurate and effective than single analysis methods in revealing the genetic mechanisms of traits.

To investigate the genetic basis of soybean plant height variation, we chose two parents with significant genetic differences in plant height, “Dongnong L13” and “Henong 60”, to construct a RIL population with 156 lines. We used simple-sequence repeat (SSR) markers for genotyping and obtained QTLs related to plant height from linkage analysis between genotypic data and phenotypic data obtained in different environments. We then performed SNP genotyping of the RIL population using gene chip technology and conducted association analysis between SNP marker genotypes and phenotypic traits. Association analysis results not only further verified the QTLs obtained by linkage analysis, but also narrowed the search for candidate genes related to plant height, which will accelerate molecular breeding to select and improve agronomic traits in soybean.

## Materials and Methods

### Plant Materials

Two soybean cultivars, “Dongnong L13” (P1) and “Henong 60” (P2), with wide genetic differences in plant height were used as parents for constructing RILs. “Dongnong L13” was bred by crossing “Heinong 40” and “Jiujiao 5640”, and “Henong 60” was bred by crossing “Beifeng 11” and “Hobbit”. The cross P1 × P2 was carried out in Harbin (E 126.63°, N 45.75°), Heilongjiang Province, China, and F_1_ seeds were harvested in 2008. In the same winter, F_1_ seeds were sowed in Yacheng City, Hainan Province (E 109.00°, N 17.50°). From 2010 to 2014, F_1_ plants were self-crossed in Harbin and Yacheng following the single-seed descent method, that is, single seeds were selected from individual plants at each generation until all individuals showed homozygous genotypes. RIL6013 containing 156 families was obtained and used for construction of genetic maps and QTL mapping.

### Field Experiment and Trait Evaluations

Experiments were carried out at four locations: Keshan (E 125.64°, N 48.25°), Harbin, Acheng (E 127.63°, N 45.82°), and Shuangcheng Cities (E 126.92°, N 45.75°). Field tests of nine environments across 4 years and four locations were carried out: Keshan (E1) in 2013, Harbin (E2) in 2014, Keshan (E3) and Harbin (E4) in 2015, Acheng (E5), Shuangcheng (E6), and Harbin (E7) in 2016, Shuangcheng (E8) in 2017, and Acheng (E9) in 2018. All plant materials in each environment were grown in three-row 3 m × 0.7 m plots in a completely randomized block design with three replications. Experimental plots were managed identically to local soybean crops.

Plant height was investigated in the field after maturity and defined as length from the cotyledon mark to the top of the main stem. Plant height of the 156 RILs was measured with a meter ruler. Plants with marginal utility were avoided during the survey, and 10 plants were randomly selected from each plot to conduct field investigations to determine plant height. The mean value of the 10 observations was taken as the observation value for the plot, and the average of the observation values of the three block replicates was used as the phenotypic data for QTL mapping.

### Statistical Analyses of Phenotypic Data

Minimum, maximum, mean, standard deviation, kurtosis, and skewness of the sample observations were calculated using the software SAS V9.21 (https://www.sas.com/en_us/home.html) and histograms of plant height frequency distribution of the RIL6013 population in the nine environments were drawn. The significance of the genotypic variance in each group of observations was then calculated using the general linear model (GLM) for analysis of variance (ANOVA). Finally, the VARCOMP command was used to estimate the variance component of the mixed linear model (MLM), and the generalized heritability of plant height in a single environment was calculated according to the following equation:

$${\text{h}}^{2}=\frac{\sigma_{\text{G}}^{2}}{\sigma_{\text{G}}^{2}{\text{+σ}}_{\text{ϵ}}^{2}}$$

(where $\sigma_{\text{G}}^{2}$ is the genotypic variance, $\sigma_{\text{ϵ}}^{2}$ is the error variance).

### SSR Marker Map and QTL Analysis

Construction of the RIL6013 map was based on the method of Ning et al. (2018). The genetic map contained 20 soybean linkage groups and 137 SSR markers covering a total genome length of 1886.80 cM. The genetic distance of each linkage group was 19.68 cM (H) – 163.67 cM (F), and the average genetic length was 94.34 cM. Each linkage group contained four to 11 markers, and the average genetic distance between the two markers was 16.13 cM.

Interval mapping (IM-ADD) and inclusive composite interval mapping (ICIM-ADD) under the .bip (QTL mapping in bi-parental populations) function built into the software QTL Icimapping V 4.1 (Wang, 2009) were used to detect additive QTLs. The Scan Step was set to 1.00 cM and the LOD threshold was set to 2.50. In addition, for ICIM-ADD, the PIN value was set to 0.001. After the QTL positioning results were obtained, they were named according to the method of McCouch (1997).

### SNP Genotyping

DNA samples extracted were used for SNP genotyping using the SoySNP660K BeadChip at Beijing Boao Biotechnology Co., Ltd. A total of 54836 SNPs across 20 chromosomes remained after quality filtering; SNP markers were excluded by minor allele frequency (MAF < 0.05), and the maximum missing sites per SNP was <10% (Belamkar et al., 2016). These SNPs were used for analysis of population structure and GWAS.

### Analysis of Population Structure

The analysis of population structure was performed using STRUCTURE V2.3.4 (Pritchard et al., 2000). For each run, the number of burn-in iterations was set to 100000, and the number of Marko Chain Monte Carlo (MCMC) was set to 100,000, while the mixed model and allele frequency correlation model were considered in the analysis. Set the K number of the subpopulations in the population from 1 to 10, and number of iterations was set to 5.To determine the best K value using STRUCTURE HARVESTER (Earl, 2012) (http://taylor0.biology.ucla.edu/structureHarvester).

### Linkage Disequilibrium Analysis

TASSEL 5.0 (Bradbury et al., 2007) was used to analyze linkage disequilibrium (LD) by analyzing r^2^ values of all pairs. The LD decay trend was analyzed using negative natural logarithm, and the physical distance of LD decay was estimated to where r^2^ dropped to 0.2.

### Genome-Wide Association Studies

mrMLM.GUI software was used to perform GWAS with the following the five methods for identifying significant QTNs: mrMLM (Wang et al., 2016), FASTmrMLM (Tamba et al., 2017), FASTmrEMMA (Wen et al., 2017), pLARmEB (Zhang et al., 2017), and ISIS EM-BLASSO (Tamba et al., 2017). The critical P value of FASTmrEMMA was set to 0.005 while the critical P value parameter of the other methods was set to 0.01, and the critical LOD value of significant QTNs was set to 3 (Wang et al., 2016). QTNs located in at least two environments or detected using two different methods were considered to be significant. The kinship matrix used in the analysis process was also calculated by the software.

### Identification of Potential Candidate Genes

QTNs were used to predict potential candidate genes based on GWAS. Genes highly expressed in stems according to the Phytozome website (https://phytozome.jgi.doe.gov) were searched for in the interval of LD decay distance when r^2^ dropped to 0.2 on both sides of the QTN. These genes were used for pathway analysis by combining gene annotation information and protein conserved domain functions from the NCBI database (https://www.ncbi.nlm.nih.gov/) and previous studies. Potential candidate genes were identified from the pathway analysis and used to identify homologous genes on the ensemble plant website (http://plants.ensembl.org/index.html) and speculate on their potential functions based on their gene ontology (GO) number (https://www.ebi.ac.uk/QuickGO/).

## Results and Analysis

### Phenotypic Variation Analysis

**Figure 1:**
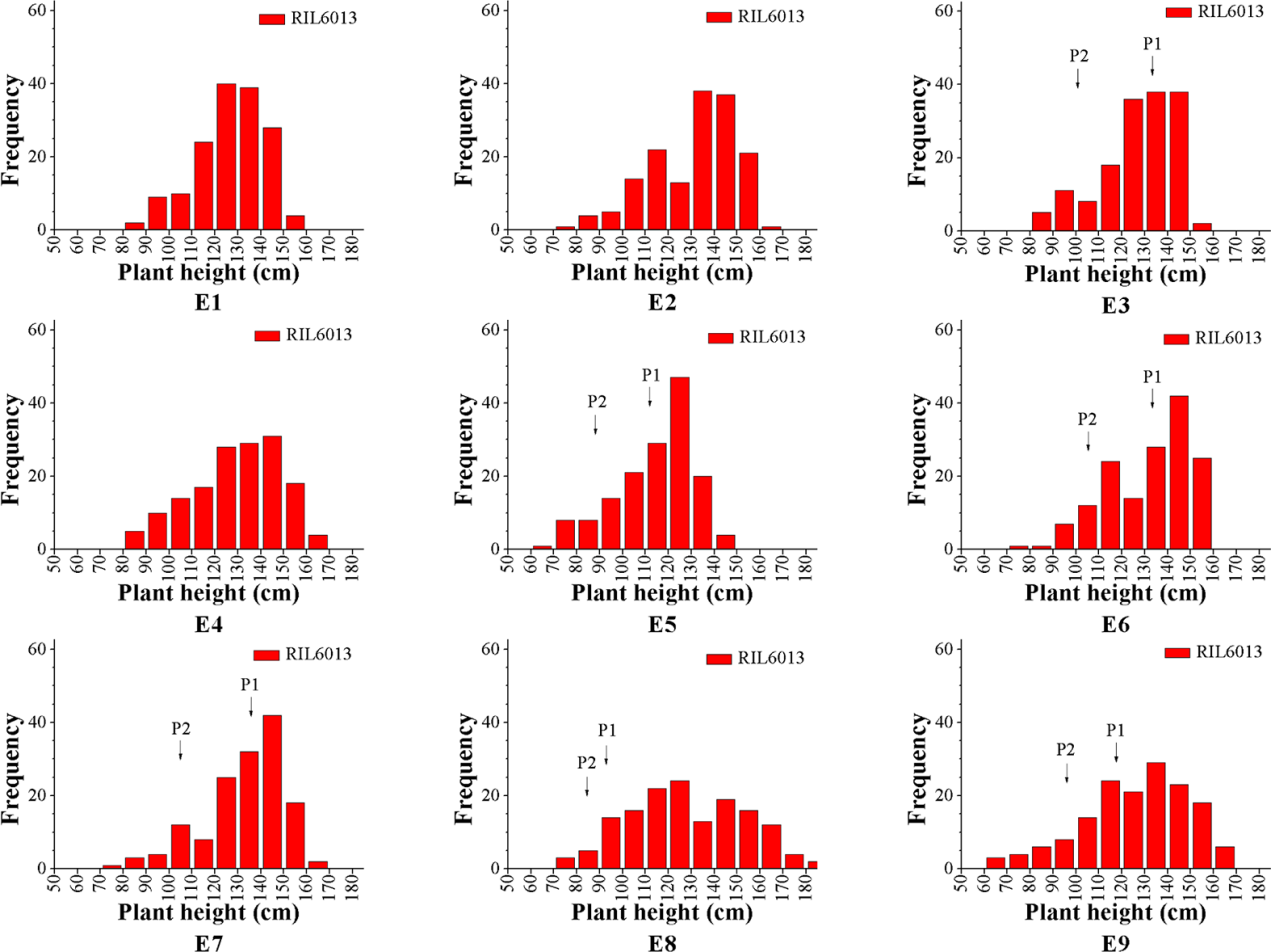
Frequency histograms of plant height in RIL6013 in population in nine environments.

The RIL6013 population showed a bimodal distribution in the nine environments, except for E8 and E6, and plant height observations generally showed a unimodal distribution. We speculate that the plant height of this population may be regulated by multiple major and minor genes (**Figure 1**). The plant height range of the population was generally higher than that of the parents, indicating a large separation within the population. Genotypic variances between lines were extremely significant (P < 0.01), and the kurtosis and skewness of statistical observations ranged from [−1, 1]. We therefore considered that this population was suitable for plant height research and QTL identification (**Table 1**).

**Table 1:** Statistical analysis of soybean plant height for parents and the RIL6013 population in nine environments.

Env.^a^	Parents^b^		RIL6013 population							
	P_1_	P_2_	Min^c^	Max^d^	Range^e^	Mean ± STD^f^	Skew	Kurt	*F* ^g^	*h^2^* ^h^
E1	–	–	77.00	144.00	67.00	117.71 ± 15.12	−0.61	−0.19	9.69**	74.30
E2	–	–	67.40	151.40	84.00	120.57 ± 18.55	−0.72	−0.21	13.12**	80.05
E3	131.00	102.00	72.80	145.80	73.00	116.98 ± 16.32	−0.78	−0.04	11.75**	78.07
E4	-	-	72.00	157.00	85.00	120.83 ± 19.14	−0.52	−0.46	13.90**	81.02
E5	114.00	88.67	59.00	135.33	76.33	104.69 ± 17.87	−0.76	−0.21	12.76**	79.67
E6	135.67	109.84	65.33	149.00	83.67	122.41 ± 18.12	−0.67	−0.42	12.80**	79.82
E7	137.50	107.00	63.50	152.00	88.50	122.88 ± 17.77	−0.95	0.54	11.47**	77.59
E8	91.33	84.00	66.50	172.50	106.00	118.57 ± 24.73	0.08	−0.74	23.81**	88.61
E9	116.67	95.17	51.33	156.00	104.67	116.08 ± 23.21	−0.58	−0.10	27.30**	89.78

a Env, environment: E1, Keshan City, 2013; E2, Harbin City, 2014; E3, Keshan City, 2015; E4, Harbin City, 2015; E5, Acheng City, 2016; E6, Shuangcheng City, 2016; E7, Harbin City, 2016; E8, Shuangcheng City, 2017; E9, Acheng City, 2018.

b Parents: P_1_, female cultivar “Dongnong L13”; P_2_, male cultivar “Henong 60”.

c Min, minimum observed value among samples.

d Max, maximum observed value among samples.

e Range, difference between maximum and minimum value.

f Mean ± standard deviation of the observed values.

g F-value for genotypic variation; **extremely significant difference at level of P < 0.01.

h h^2^, Broad-sense heritability of plant height in a single environment.

The coefficient of variation (CV) for plant height was between 12.84% and 20.86%. The range of plant heights in RIL6013 demonstrated that plant height variation is large. Mean heights of the population under E4, E6, and E7 were higher than those under the other six environments, and the CVs in E8 and E9 were larger than those in the other seven environments (**Table 1**). This suggested that different QTLs might be detected in different environments.

From a breeding point of view, except in E8, the plant height of the parents fell within the range of [Mean−STD, Mean+STD] for the population, indicating that the population has strong transgressive heterosis and plant height breeding goals will have a larger choice within the population.

The heritability of RIL6013 was between 74.30% and 89.78% in a single environment (**Table 1**). This high heritability indicates that the genetic variance is superior to the error variance, and RIL6013 is suitable for high-quality selection of plant height in soybean.

Correlation analysis revealed a strong correlation between the various environments (**Table 2**). The ANOVA results showed extremely significant differences (P<0.01) between the genotypes of this population, and also significant differences between the various environments. There were significant differences between genotypes, environment, and the genotype-by-environment interaction (**Table 3**), indicating that soybean plant height is highly influenced by all of these factors.

**Table 2:** Correlation analysis of nine environments.

	E1	E2	E3	E4	E5	E6	E7	E8	E9
E1	1								
E2	0.731^**^	1							
E3	0.055	0.048	1						
E4	0.062	0.044	0.091	1					
E5	−0.053	-0.046	0.002	−0.040	1				
E6	0.012	−0.007	0.023	−0.046	0.681^**^	1			
E7	−0.066	−0.105	0.035	0.015	0.616^**^	0.669^**^	1		
E8	−0.231^**^	−0.112	−0.115	−0.060	0.070	−0.053	0.005	1	
E9	−0.217^**^	−0.121	−0.093	−0.089	−0.147	−0.114	−0.125	0.688^**^	1

**Extremely significant difference at level of P < 0.01.

**Table 3:** Joint ANOVA of plant height in RIL6013 in multiple environments.

	DF	SS	MS	F	Pr > F
Environment	8	106218.84	13277.36	170.54^**^	<0.0001
Genotype	155	240233.10	1549.89	19.91^**^	<0.0001
Environment*Genotype	1219	1343337.47	1102.00	14.15^**^	<0.0001
Error	2765	215274.53	77.86		

**Extremely significant difference at level of P < 0.01.

### QTL Mapping of the Plant Height

We detected 48 plant height QTLs (**Table S1**), distributed across the 20 soybean linkage groups. One (I) to five (M) QTLs mapped to each linkage group with LOD values between 2.54 and 13.46, and each QTL explained between 0.55% and 19.55% of the phenotypic variation. Four QTLs (*qPH-C1-1*, *qPH-M-1*, *qPH-F-1*, *qPH-L-1*) explaining >10% phenotypic variation can be considered the major QTLs controlling plant height.

Twelve QTLs were detected by both IM and ICIM methods and were distributed on 10 linkage groups A2, B1, C1, C2, D1a, D1b, F, H, L, and N (**Table S2**). LOD values were between 2.54 and 13.46, and the phenotypic variation explained (PVE) ranged from 1.45% to 19.55%. The additive effects of *qPH-F-1* detected in the E8 environment were negative, indicating that the allele derived from the male parent “Henong 60” increased plant height. The additive effects of the remaining QTLs were positive, indicating that the allele derived from the female parent “Dongnong L13” increase plant height.

We identified 21 QTLs in two or more environments, distributed over 14 linkage groups except D1b, A1, C2, B2, E, and I (**Table S3**). LOD values were 2.58 to 13.46, and the PVE ranged from 0.55% to 14.83%. Additive effects of *qPH-K-2* detected in E1 and E2, *qPH-F-1* detected in E8, and *qPH-J-1* detected in E1 and E2 were negative, indicating the genes causing greatest effect were from the male parent; for the remaining QTLs, the genes making the greatest contribution were from the female parent.

**Figure 2:**
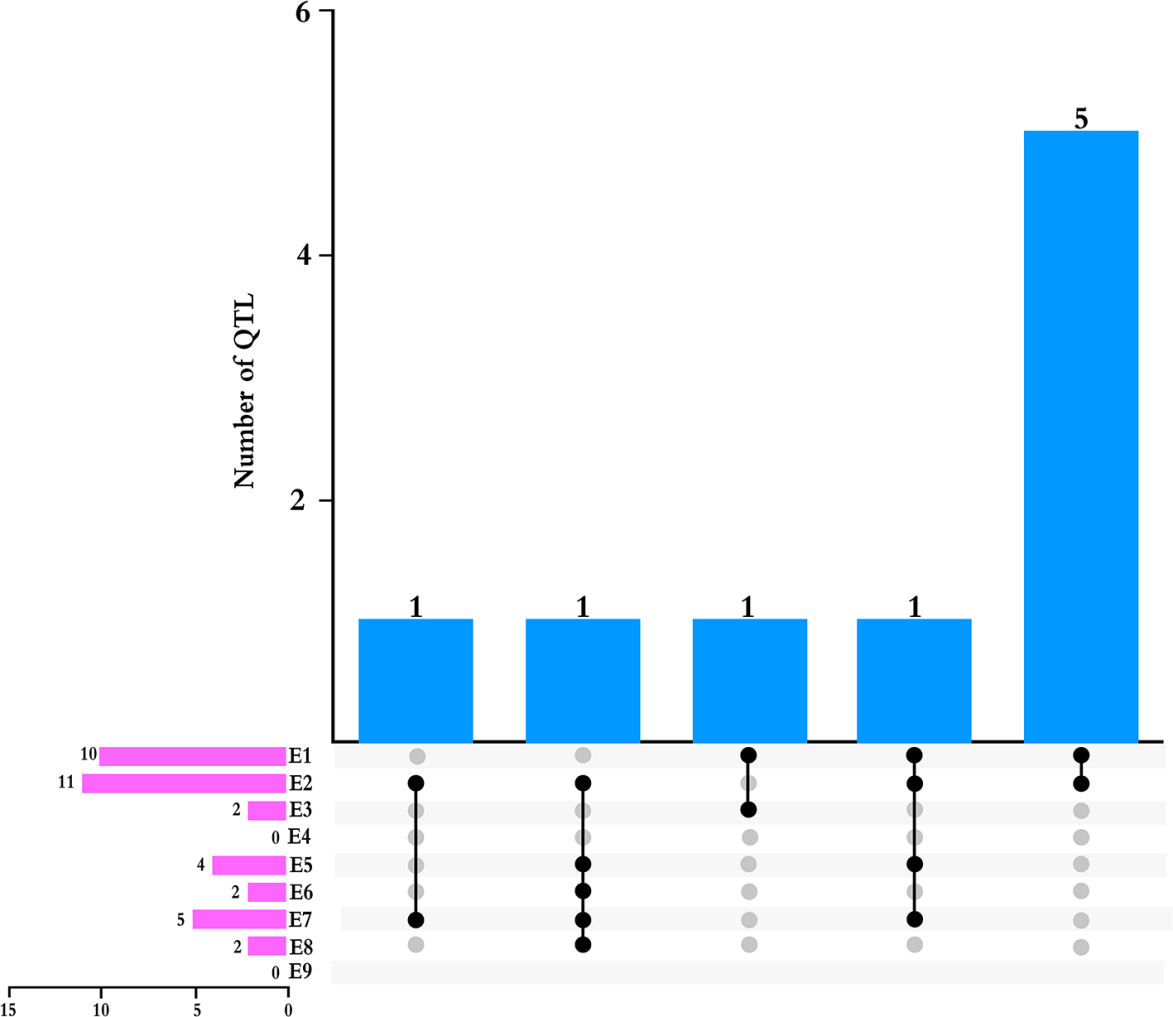
Total numbers of QTLs detected across multiple environments and by multiple methods. Pink bars indicated the number of QTLs detected in each environment.

We detected nine QTLs across multiple environments and using multiple methods (**Table 4**): E2 and E7, one QTL (*qPH-A2-1*); E1 and E3, one QTL (*qPH-N-2*); E1 and E2, five QTLs (*qPH-B1-1*, *qPH-C1-1*, *qPH-D1a-3*, *qPH-H-1*, and *qPH-L-1*); E1, E2, E5, and E7, one QTL (*qPH-D1a-2*); E2, E5, E6, E7, and E8, one QTL (*qPH-F-1*) (**Figure 2**). *qPH-A2-1* was detected in the A2 linkage group, with LOD values from 2.97 to 3.13, PVE ranging from 1.13% to 6.93%, and a positive additive effect indicating that the dominant gene was derived from the female parent. *qPH-N-2* was detected in the N linkage group with LOD value of 2.92 to 3.14 and PVE ranging from 1.12% to 1.75%, in which the gene with greatest contribution to the QTL under E1 was derived from the male parent while that with the greatest contribution under E3 was derived from the female parent. *qPH-B1-1* was detected in the B1 linkage group, with LOD value of 4.86 to 7.27, PVE ranging from 2.12% to 9.79%, and the dominant gene derived from the female parent. *qPH-C1-1* was detected in the C1 linkage group, with LOD value of 3.79 to 13.46, PVE ranging from 1.91% to 19.55%, and the dominant gene derived from the female parent. *qPH-D1a-3* was detected in the D1a linkage group, with LOD value of 2.64 to 8.35, PVE ranging from 1.60% to 9.38%, and the dominant gene derived from the female parent. *qPH-H-1* was detected in the H linkage group, with LOD value of 4.84 to 7.00, PVE from 1.02% to 7.04%, and the dominant gene derived from the female parent. *qPH-L-1* was detected in the L linkage group, with LOD value of 4.58 to 8.20, PVE ranging from 3.02% to 14.00%, and the dominant gene derived from the female parent. *qPH-D1a-2* was detected in the D1a linkage group, with LOD value of 2.83 to 5.27, PVE from 2.52% to 5.71%, and the dominant gene derived from the female parent. *qPH-F-1* was detected in the F linkage group, with LOD value of 3.29 to 6.97, and PVE ranging from 1.14% to 10.72%; the additive effect in E2 and E8 was negative, indicating that the dominant gene was derived from the male parent.

**Table 4:** Stably expressed QTLs identified by multiple methods and in multiple environments.

QTL	Environment	Chromosome	Left marker	Right marker	LOD^a^	PVE(%)^b^	Add^c^	Method
*qPH-A2-1*	E2,E7,E7	8	Satt119	Sat_406	2.97,3.13,3.13	1.13,4.31,6.93	13.12,16.38,16.38	IM,IM,ICIM
*qPH-B1-1*	E1,E2,E2	11	Satt197	Sat_123	5.15,7.27,4.86	3.99,2.12,9.79	12.07,15.44,10.64	IM,IM,ICIM
*qPH-C1-1*	E1,E2,E2	4	Satt713	Satt565	3.79,13.46,10.84	1.91,2.74,19.55	6.95,15.47,12.80	IM,IM,ICIM
*qPH-D1a-2*	E1,E2,E5,E7,E5	1	Sat_413	Sat_160	2.83,5.27,4.75,3.65,4.75	4.54,2.52,2.64,5.71,2.64	12.18,16.28,17.27,17.56,17.27	IM,IM,IM,IM,ICIM
*qPH-D1a-3*	E1,E2,E1,E2	1	Satt515	AZ302047	7.91,8.35,4.90,2.64	3.18,1.60,9.38,3.22	9.25,11.92,6.33,5.14	IM,IM,ICIM,ICIM
*qPH-F-1*	E2,E5,E6,E7,E8,E5,E6,E7,E8	13	Sat_039	Sat_417	6.97,3.29,5.16,4.31,3.33,3.29,5.16,3.58,3.33	1.14,3.02,2.28,5.22,3.49,3.02,2.28,7.69,10.72	−9.55,15.15,16.14,14.20,−9.59,15.15,16.14,13.27,−9.59	IM,IM,IM,IM,IM,ICIM,ICIM,ICIM,ICIM
*qPH-H-1*	E1,E2,E1	12	Satt181	Satt434	7.00,6.50,4.84	2.33,1.02,7.04	8.41,10.05,5.82	IM,IM,ICIM
*qPH-L-1*	E1,E1,E2	19	Sat_195	Satt448	4.67,4.58,8.20	3.02,14.83,14.00	13.19,11.54,16.03	IM,ICIM,ICIM
*qPH-N-2*	E1,E3,E3	3	Satt584	Satt660	3.14,2.92,2.92	1.12,1.45,1.75	−8.28,9.67,9.67	IM,IM,ICIM

a LOD, logarithm of odds.

b PVE, phenotypic variation explained by QTL.

c Add, contribution of parents to the additive effect.

### Population Structure

**Figure 3:**
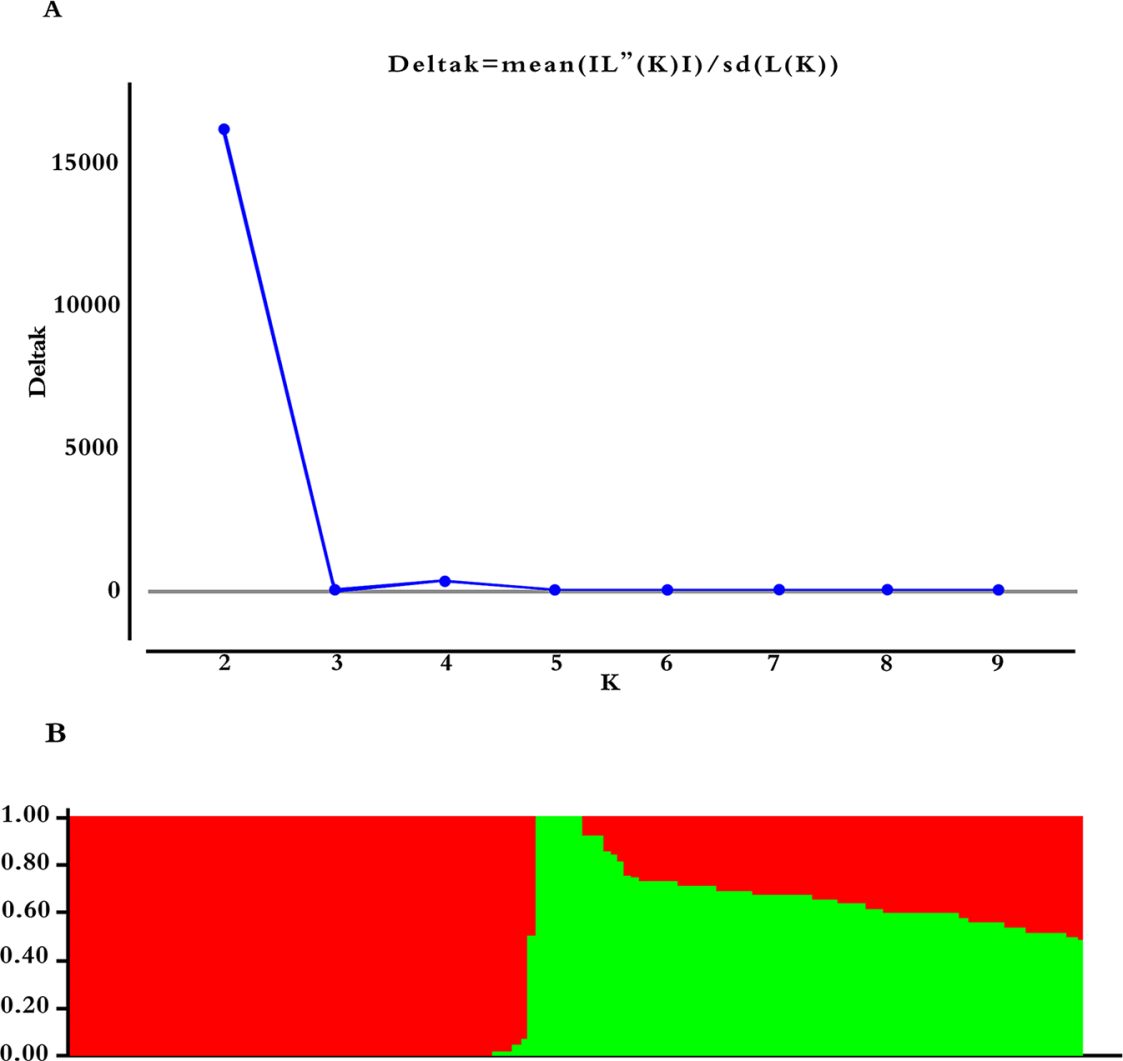
Population structure based on 5,000 SNPs distributed across 20 chromosomes. **(A)** Plot of ΔK calculated for K = 1–10. **(B)** Population structure (K = 2); the areas of the two colors (green and red) illustrate the proportion of each subgroup.

Selected 5000 of the 54836 with more polymorphic SNP markers (Pritchard et al., 2003), which were randomly distributed on 20 soybean chromosomes. Calculating ΔK using STRUCTURE 2.3.4 (**Figure 3A**, K = 1–10), revealing two subgroups (K = 2) based on the ΔK value (**Figure 3B**). These two subgroups contain 64 (46.04%) and 75 (53.96%) lines, respectively.

### Linkage Disequilibrium Analysis

Because we used a bi-parental population, the LD decay distance was much longer than that in the natural population. As shown in **Figure S1**, r^2^ decreased gradually with increased distance, and the LD decay distance was estimated at 950 kb when r^2^ was 0.2. As this distance is too large, we determined the range for potential candidate genes according to the region showing the fastest LD decay. LD decayed fastest within 200 kb, and then gradually slowed down, so we searched for potential candidate genes in the interval of 100 kb on either side of each QTN.

### QTN Location by Multi-Locus GWAS Methods

**Figure 4:**
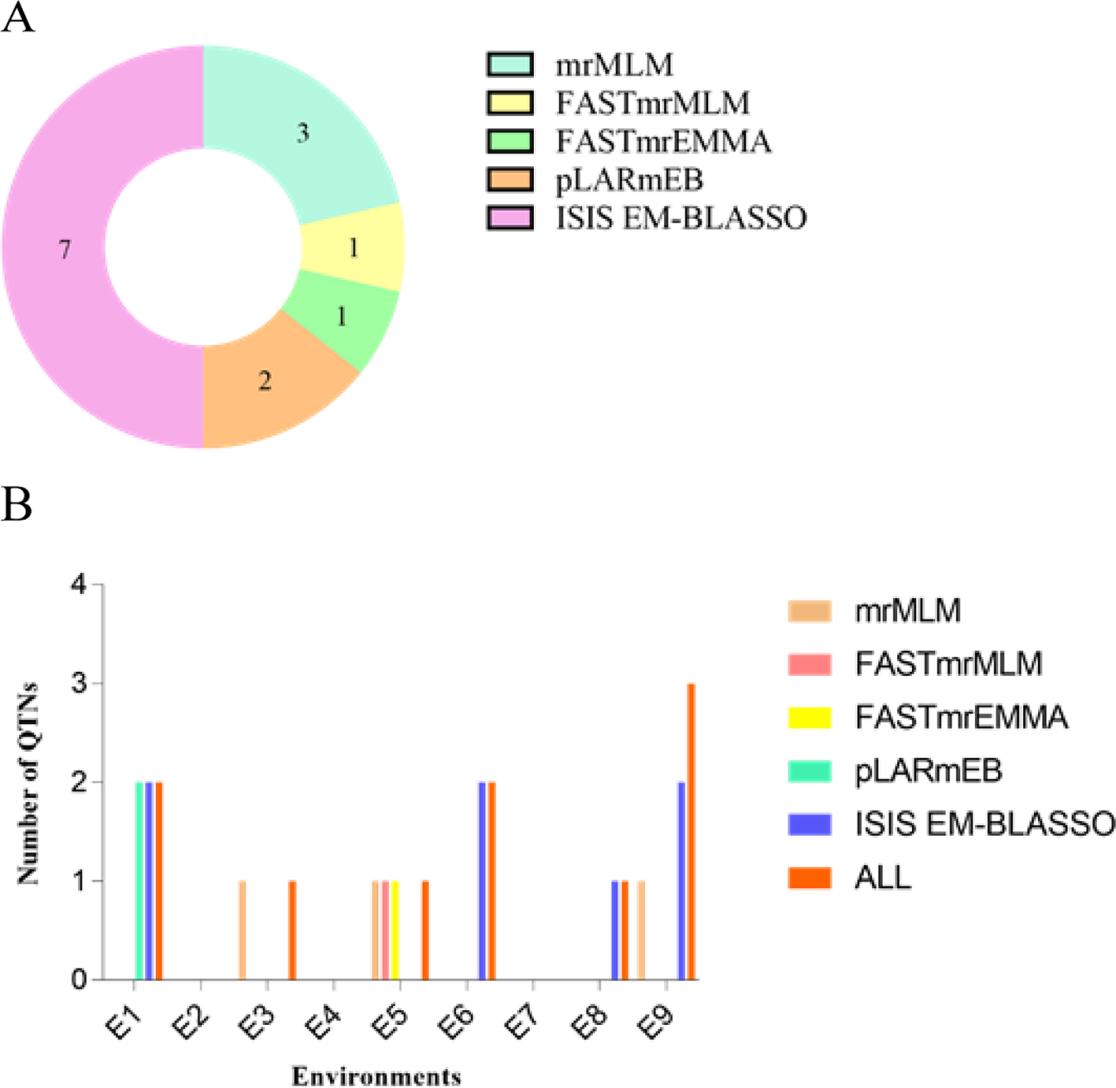
**(A)** Total number of significant QTNs detected by five methods. **(B)** Total number of significant QTNs detected in nine environments using five methods.

We used mrMLM, FASTmrMLM, FASTmrEMMA, pLARmEB, and ISIS EM-BLASSO to detect 10 QTNs (**Table 5**), and the five methods detected three, one, one, two, and seven QTNs, respectively (**Figure 4A**). We detected two, one, one, two, one, and three QTNs in environments E1, E3, E5, E6, E8, and E9, respectively (**Figure 4B**). No significant QTN was identified in environments E2, E4, or E7.

**Table 5:** QTNs identified by multiple methods.

Environment	Method^a^	Marker	Chromosome	Position (bp)	QTN effect	LOD score	r2 (%)^b^
E6	5	AX-157411267	2	14266435	−9.02	3.87	9.76
E3	1	AX-157176525	4	10948200	6.82	3.25	16.47
E6	5	AX-157526374	4	2455146	5.94	3.76	8.86
E1	4,5	AX-157388309	7	35573836	6.56,6.56	4.93,5.77	14.25,16.42
E9	5	AX-157136514	8	46855033	−6.50	3.38	6.11
E9	5	AX-157144941	9	38573690	−8.21	4.19	10.65
E5	1,2,3	AX-157278476	13	11184314	−8.61,−7.25,−14.48	4.49,3.46,4.44	16.93,12.08,11.98
E9	1	AX-157296110	13	42895901	10.15	3.55	16.30
E8	5	AX-157143002	16	36971410	9.90	3.42	9.44
E1	4,5	AX-117466184	20	46210693	7.04,7.04	3.68,4.05	9.16,10.55

a mrMLM, FASTmrMLM, FASTmrEMMA, pLARmEB, and ISIS EM-BLASSO are indicated by 1 to 5, respectively.

b r2 (%), proportion of total phenotypic variation explained by each QTN.

We detected three QTNs using multiple methods (**Table 6**, **Figure 4B**). The pLARmEB and ISIS EM-BLASSO methods detected a single QTN (AX-157388309) on chromosome 7, with LOD values ranging from 4.93 to 5.77 and PVE of 14.25% to 16.42%, and a QTN (AX-117466184) located on chromosome 20, with LOD value of 3.68 to 4.05 and PVE ranging from 9.16% to 10.55%. Both QTN effects were positive. The mrMLM, FASTmrMLM, and FASTmrEMMA methods detected a single QTN (AX-157278476) located on chromosome 13, with LOD value ranging from 3.46 to 4.49 and PVE ranging from 11.98% to 16.93%; the QTN effect was negative. The ISIS EM-BLASSO method detected the largest number of QTNs located in multiple environments, and the QTN effects detected by various methods were consistent (both positive or negative).

**Table 6:** Stably expressed QTNs identified by multiple methods.

Environment	Method^a^	Marker	Chromosome	Position (bp)	QTN effect	LOD score	r2 (%)^b^
E1	4,5	AX-157388309	7	35573836	6.56,6.56	4.93,5.77	14.25,16.42
E5	1,2,3	AX-157278476	13	11184314	−8.61,−7.25,−14.48	4.49,3.46,4.44	16.93,12.08,11.98
E1	4,5	AX-117466184	20	46210693	7.04,7.04	3.68,4.05	9.16,10.55

a mrMLM, FASTmrMLM, FASTmrEMMA, pLARmEB, and ISIS EM-BLASSO were indicated by 1 to 5, respectively.

b r2 (%), proportion of total phenotypic variation explained by each QTN.

### Potential Candidate Gene Determination

**Figure 5:**
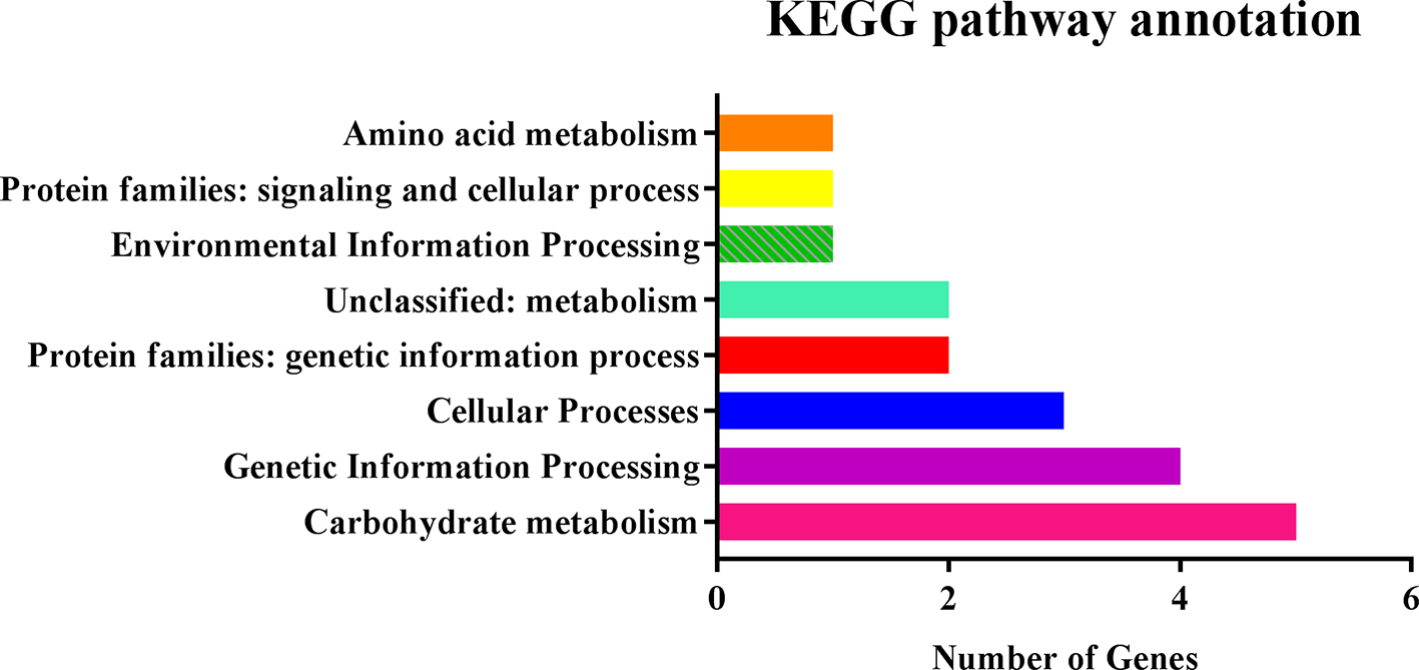
KEGG pathway annotation for 19 candidate genes.

We searched for potential candidate genes within 100-kb intervals on both sides of four QTNs, AX-157278476, AX-157296110, AX-157176525, and AX-157526374, which were repeatedly identified by linkage analysis and GWAS. Five genes were in the interval 11.08–12.08 Mb and 25 genes in the interval 42.80–43.00 Mb on chromosome 13, and three genes were in the interval 10.84–11.04 Mb and 29 in the interval 2.36–2.56 Mb on chromosome 4. We identified a total of 62 genes, of which 56 were expressed in stems and 24 were highly expressed in stems according to the Phytozome website. We used these 56 genes for pathway analysis, of which 19 (33.9%) were annotated in 16 pathways in the KEGG database (http://www.kegg.jp/) (**Table 7**, **Figure 5**). Through the ensemble plant website, we identified homologs of the 56 candidate genes in *Arabidopsis thaliana*, and predicted their possible functions (**Table 8**).

**Table 7:** Details of 19 candidate genes annotated in the KEGG database.

QTN name	Gene name	Chromosome	Position	KO number	Annotation
AX-157296110	*Glyma.13G334200.1*	chr13	42794878..42801645	K12881	THOC4; THO complex subunit 4
**AX-157296110**	***Glyma.13G334300.1***	**chr13**	**42807703..42808635**	**K11251**	**H2A; histone H2A**
**AX-157296110**	***Glyma.13G334500.1***	**chr13**	**42815054..42819704**	**K08678**	**UXS1; UDP-glucuronate decarboxylase [EC:4.1.1.35]**
**AX-157296110**	***Glyma.13G334800.1***	**chr13**	**42840520..42845919**	**K10575**	**UBE2G1; ubiquitin-conjugating enzyme E2 G1 [EC:2.3.2.23]**
AX-157296110	*Glyma.13G335000.1*	chr13	42851863..42856533	K14833	NOC2; nucleolar complex protein 2
AX-157296110	*Glyma.13G335600.1*	chr13	42916206..42918785	K10355	ACTF; actin, other eukaryote
AX-157296110	*Glyma.13G335800.1*	chr13	42925815..42927692	K12471	EPN; epsin
AX-157296110	*Glyma.13G336200.1*	chr13	42957851..42960820	K01803	TPI; triosephosphate isomerase (TIM) [EC:5.3.1.1]
AX-157526374	*Glyma.04G029000.1*	chr04	2354851..2358499	K07056	rsmI; 16S rRNA (cytidine1402-2'-O)-methyltransferase [EC:2.1.1.198]
AX-157526374	*Glyma.04G029400.1*	chr04	2378504..2382290	K03064	PSMC6; 26S proteasome regulatory subunit T4
AX-157526374	*Glyma.04G029700.1*	chr04	2405521..2408263	K20535	MPK1_2; mitogen-activated protein kinase 1/2 [EC:2.7.11.24]
AX-157526374	*Glyma.04G029800.1*	chr04	2411114..2416765	K12402	AP4M1; AP-4 complex subunit mu-1
**AX-157526374**	***Glyma.04G030000.1***	**chr04**	**2429647..2437011**	**K10572**	**IPPK; inositol-pentakisphosphate 2-kinase [EC:2.7.1.158]**
AX-157526374	*Glyma.04G030300.1*	chr04	2461743..2467168	K00975	glgC; glucose-1-phosphate adenylyltransferase [EC:2.7.7.27]
**AX-157526374**	***Glyma.04G030400.1***	**chr04**	**2466301..2470762**	**K19355**	**MAN; mannan endo-1,4-beta-mannosidase [EC:3.2.1.78]**
AX-157526374	*Glyma.04G030500.1*	chr04	2481992..2488564	K00611	OTC; ornithine carbamoyltransferase [EC:2.1.3.3]
AX-157526374	*Glyma.04G030700.1*	chr04	2493579..2495558	K01726	GAMMACA; gamma-carbonic anhydrase [EC:4.2.1.-]
AX-157526374	*Glyma.04G030800.1*	chr04	2497398..2501640	K01726	GAMMACA; gamma-carbonic anhydrase [EC:4.2.1.-]
AX-157526374	*Glyma.04G031600.1*	chr04	2525754..2530508	K01247	alkA; DNA-3-methyladenine glycosylase II [EC:3.2.2.21]

Bold font indicates genes that we propose correlate with plant height in soybean.

**Table 8:** Annotation information of homologous genes in *Arabidopsis thaliana*.

Gene name	GO pathway annotation information	Homologous gene	Homologous gene description
***Glyma.13G035900***	**GO:0004672**	***AT1G21250***	**Wall-associated receptor kinase 1**
*Glyma.13G036000*	GO:0006886	*AT1G21380*	TOM1-like protein 3
*Glyma.13G334200*	GO:0003676	*AT5G59950*	RNA-binding (RRM/RBD/RNP motifs) family protein
*Glyma.13G334500*	GO:0003854	*AT2G28760*	UDP-XYL synthase 6
*Glyma.13G334700*	–	*AT3G46450*	SEC14 cytosolic factor family protein / phosphoglyceride transfer family protein
*Glyma.13G334800*	–	*AT3G46460*	UBC13
*Glyma.13G335100*	–	*AT5G59350*	Transmembrane protein
*Glyma.13G335300*	–	*AT2G28840*	Putative E3 ubiquitin-protein ligase XBAT31
*Glyma.13G336000*	–	*AT1G07590*	Pentatricopeptide repeat-containing protein At1g07590, mitochondrial
*Glyma.13G336500*	GO:0031047	*AT1G13790*	Factor of DNA methylation 4
*Glyma.04G029400*	GO:0006281	*AT1G45000*	26S proteasome regulatory subunit S10B homolog B
*Glyma.04G029500*	–	*AT1G75400*	RING/U-box superfamily protein
*Glyma.04G029700*	GO:0005524	*AT1G10210*	MPK1
*Glyma.04G029700*	GO:0005524	*AT1G59580*	Mitogen-activated protein kinase
*Glyma.04G029900*	–	*AT1G19650*	Phosphatidylinositol/phosphatidylcholine transfer protein SFH4
***Glyma.04G030000***	**GO:0005524**	***AT1G22100***	**Inositol-pentakisphosphate 2-kinase**
*Glyma.04G030300*	GO:0009058	*AT1G27680*	Glucose-1-phosphate adenylyltransferase large subunit 2, chloroplastic
*Glyma.04G031600*	GO:0006284	*AT1G75230*	DNA glycosylase superfamily protein
*Glyma.04G031800*	–	*AT1G75210*	Cytosolic IMP-GMP specific 5-nucleotidase, putative

Bold font indicates genes that we propose correlate with plant height in soybean.

## Discussion

There are two parents, Dongnong L13 (110 cm) and Heongong 60 (70 cm), with significant differences in plant height, were distributed among 156 families. At the same time, the plant height observations of each family in the population showed a unimodal distribution, indicating that the plant height is a typical quantitative trait and controlled by multiple genes. The variation of plant height among the families in the population was large, and the genetic basis was rich. The range of RIL6013 is large than population conducted by “Minsoy” and “Noir 1”, indicated this population is more suitable for QTL mapping. At the same time, the results of the *F* test showed that the differences between the families in each environment were extremely significant (P < 0.01), also indicated that the population is suitable for analysis of variance and QTL mapping.

There is increasing research on QTLs related to soybean yield traits employing different methods. Most previous studies were single-environment analyses, affected by loss of related QTLs and data loss when using high LOD values or false positive detection of QTLs when using low LOD values. There are many detection methods for QTL mapping of soybean traits, including single marker analysis (SMA), interval mapping (IM), composite interval mapping (CIM), and inclusive complete interval mapping (ICIM). SMA is generally used in the case of few molecular markers, and genetic map construction is difficult. There are limitations on the development of QTL research using SMA (Haley and Knott, 1992), and genetic effects of QTLs and markers between the linkage distance cannot be distinguished. IM was first proposed by Lander and Botstein (1989). The possible location of QTLs can be inferred by marker interval, and the number of individuals required for the method is small. However, linkage between QTLs easily creates false positives, resulting in low detection efficiency of QTLs. Zeng (1993) proposed a CIM method, which introduces molecular markers closely linked to other QTLs as background genetic control, thereby reducing the residual variance, eliminating the influence of other QTLs, and improving the accuracy of QTL mapping. However, this method cannot be used in complex situations such as those involving epistasis or environment interactions. Compared with CIM, Wang (2009) proposed that ICIM simplifies the process of controlling genetic background variation and improves the detection efficiency of QTLs (Li H. et al., 2010). We used both IM and ICIM to effectively detect QTL intervals accurately in this study.

**Figure 6:**
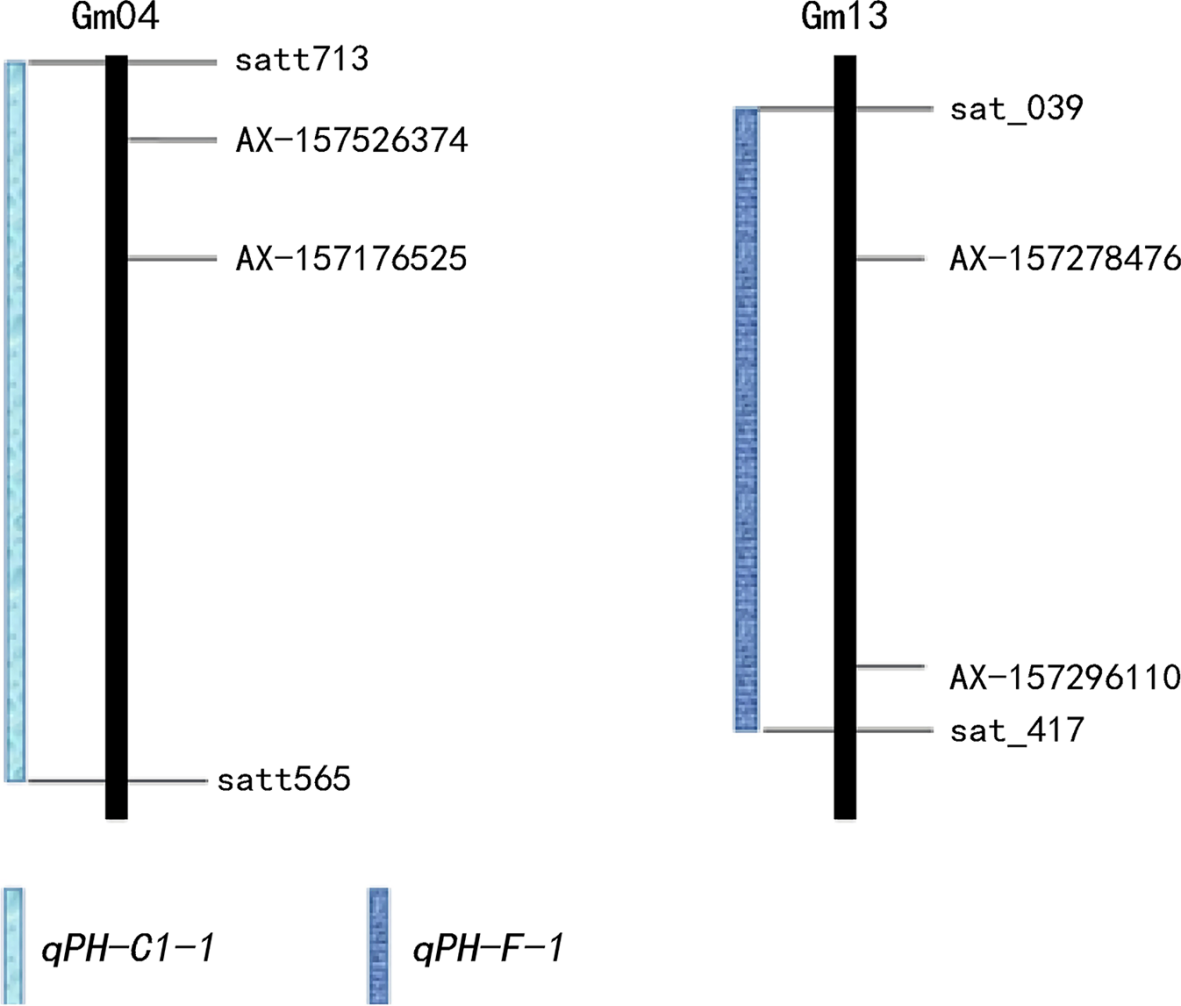
Details of overlapping loci on two chromosomes.

GWAS are effective methods for analyzing the genetic basis of complex traits in natural plant populations. Meanwhile, linkage analysis of segregating populations can effectively eliminate false positives, a defect of GWAS, but the results of linkage analysis usually have large intervals making it harder to find target genes. The four multi-locus GWAS methods can accurately map QTNs, and using two methods to verify each other can improve the accuracy of mapping. Analysis of multi-year and multi-environment data improves the accuracy of QTL position and effect estimation (Jansen et al., 1995) and is conducive to searching for stable QTLs. We also used multi-locus GWAS to detect QTNs related to soybean plant height. Linkage analysis based on multiple methods and environments detected nine QTLs (**Table 4**), while GWAS analysis detected 10 QTNs (**Table 5**). These included *qPH-F-1* located on chromosome 13 at 1.91–43.31 Mb; AX-157278476 and AX-157296110 located at 11.11 Mb and 42.90 Mb on chromosome 13, respectively; *qPH-C1-1* located at 0.55–46.13 Mb on chromosome 4; and AX-157526374 and AX-157176525 at 2.46 Mb and 10.95 Mb on chromosome 4, respectively (**Figure 6**, **Table S4**). Use of two methods not only narrowed the search range for candidate genes, but also supported the reliability of our results.

The population structure is an important factor leading to false positives in the results of association analysis, and the population structure is evaluated to determine the statistical model used. In statistics, confounding factors refer to irrelevant variable that are related to both dependent variables and independent variables (Miller, 2005). The population structure variables are confounding factors and are related to genotypes and phenotypes. At the same time, many traits are directly related to the subsets. There are differences in the phenotype and genotype between the families of the RIL population, so it is necessary to analyze the population structure of the RIL population. We performed multi-locus GWAS by a model removed Q, and the results were significantly different from the model Q+K (**Table S5**), indicating that the population structure has a great influence on the GWAS, so we adopted the model Q+K.

There is a close relationship between quantitative traits and environment. Plant height is a typical quantitative trait, and also affects soybean yield. The interaction between plant height and environmental conditions is an important factor affecting soybean yield. Huang et al. (1997) reported that QTLs are sensitive to environmental performance and there are large differences in QTL detection between different environments, but QTLs for different traits have different stability, and those with high heritability are more easily detected across different environments. Fulton et al. (1997) believe that QTLs that detect higher effect values in a single environment are not as efficient as those detected in multiple environments. Among the QTNs mapped by GWAS, three (AX-157388309, AX-157278476, and AX-117466184) that could be located by multiple methods explained phenotypic variation ranging from 9.16% to 16.93%. These stable main QTNs can be used for fine mapping and related research on marker-assisted selection.

Song et al. (2004) proposed the soybean genetic map GmComposite2003 based on the SoyBase database (https://www.soybase.org/) and results of recent studies, covering 20 chromosomes of soybean, with a total length of 2562.28 cM, and including 3334 markers. We used this as a reference map for integrating the results of this experiment. We detected nine soybean plant height QTLs using multiple methods and in multiple environments, and eight of these QTLs—*qPH-D1a-2* (Lark et al., 1995; Chen et al., 2007; Hu et al., 2013), *qPH-D1a-3* (Hu et al., 2013), *qPH-N-2* (Kabelka et al., 2004), *qPH-C1-1* (Lee et al., 1996; Kim et al., 2012), *qPH-A2-1* (Lee et al., 1996), *qPH-B1-1* (Lee et al., 1996; Sun et al., 2006; Gai et al., 2007; Sun et al., 2012), *qPH-F-1* (Lee et al., 1996; Kabelka et al., 2004; Reinprecht et al., 2006; Gai et al., 2007; Josie et al., 2007; Li D. et al., 2010; Lee et al., 2015; Yao et al., 2015; Yao et al., 2015; Zhang et al., 2018b), and *qPH-L-1* (Lark et al., 1995; Lee et al., 1996; Li et al., 2008))—showed inclusion within or overlap with chromosomal regions of QTLs detected in previous studies (**Table 9**). *qPH-H-1* detected in E1 and E2 on chromosome 12 is not reported on the genetic map. We consider this a newly discovered QTL with PVE ranging from 1.02% to 7.04%. The 10 QTNs located in this study are not overlapping with or close to previously described regions, and represent newly located QTNs, which can explain phenotypic variation of 6.11% to 16.93%.

**Table 9:** QTLs located in previous studies.

QTL	Chr	Marker interval	Reference
qPH-D1a-2	1	Sat_413-Sat_160	Chen et al., 2007; Hu et al., 2013; Lark et al., 1995
qPH-D1a-3	1	satt515-AZ302047	Hu et al., 2013
qPH-N-2	3	Satt584-Satt660	Kabelka et al., 2004
qPH-C1-1	4	Satt713-satt565	Kim et al., 2012; Lee et al., 1996
qPH-A2-1	8	satt119-sat_406	Lee et al., 1996
qPH-B1-1	11	satt197-sat_123	Gai et al., 2007; Sun et al., 2006; Lee et al., 1996; Sun et al., 2012
qPH-F-1	13	sat_039-Sat_417	Kabelka et al., 2004; Gai et al., 2007; Reinprecht et al., 2006; Yao et al., 2015; Lee et al., 2015; Josie et al., 2007; Li D et al., 2010; Zhang et al., 2018b; Lee et al., 1996; Yao et al., 2015
qPH-L-1	19	sat_195-satt448	Li et al., 2008; Lee et al., 1996; Lark et al., 1995

Based on the analysis of 19 significant genes in the KEGG pathway, we conclude that five genes may regulate the growth and development of soybean cells (**Table 7**, bold text), which in turn affects soybean plant height and yield. *Glyma.13G334500* is involved in the steroid biosynthesis process and regulates oxidoreductase activity, participates in the redox process, and is closely related to plant growth. *Glyma.04G030000* is involved in the binding of ATP and specifically binds to 5-adenosine triphosphate (Ives et al., 2000). *Glyma.04G030400* is involved in all carbohydrate pathways in the cell. *Glyma.13G334300* is involved in the biosynthesis of histone H2A, which plays an important role in regulating plant flowering, growth and development, immune response, mitosis, and DNA repair (Berriri et al., 2016). *Glyma.13G334800* regulates the production of ubiquitin ligase, which plays an important role in plant growth and development and stress regulation, including plant height and seed weight (Liu et al., 2013).These genes are all thought to be closely related to the stem growth of soybean plants.

We identified 19 genes in Arabidopsis homologous to the 56 candidate soybean genes, two of which may be associated with soybean plant height (**Table 8**). *Glyma.13G035900* appears in the GO: 0004672 pathway. The homologous gene in Arabidopsis is *AT1G21250*, which regulates the synthesis of cell wall receptor kinase and controls cell wall elongation, which may be related to plant stem growth. *Glyma.04G030000* appears in the GO: 0005524 pathway; the homologous gene in Arabidopsis is *AT1G22100*, which is involved in the synthesis of ATP and is involved in cell growth and development.

## Summary

We detected nine significant QTLs by linkage analysis and 10 QTNs using five multi-locus GWAS methods. Combining the two analysis methods, we obtained four significant QTNs and predicted five candidate genes closely related to soybean plant height around these four QTNs. We identified 19 homologous genes in Arabidopsis, two of which may regulate plant height and development.

## Data Availability Statement

All datasets generated for this study are included in the article/**Supplementary Material**. 

## Author Contributions

W-XL and HN conceived and designed the experiments. YF, QD, XL, ZQ, YW, XT, JS, JW, CY, and SJ performed the field experiments. SL and ZT performed the genome sequencing. YF, KZ, and HN analyzed and interpreted the results. YF and HN drafted the manuscript, and all the authors contributed to manuscript revision. WL provided laboratory conditions.

## Funding

The authors gratefully acknowledge the financial support for this study provided by grants from the National Key Research and Development Program of China (2017YFD0101303-6) to W-XL and Project of Research and Development on Applied Technology of Harbin in Heilongjiang Province (2017RAXXJ019) to HN.

## Conflict of Interest

The authors declare that the research was conducted in the absence of any commercial or financial relationships that could be construed as a potential conflict of interest.

## Supplementary Material

The Supplementary Material for this article can be found online at: https://www.frontiersin.org/articles/10.3389/fpls.2020.00009/full#supplementary-material
