# Supplementary figures and images for: Linkage Analysis and Multi-Locus Genome-Wide Association Studies Identify QTNs Controlling Soybean Plant Height
Source: Front Plant Sci. 2020 Feb 14;11:9. doi: 10.3389/fpls.2020.00009 (PMC7033546; doi:10.3389/fpls.2020.00009)

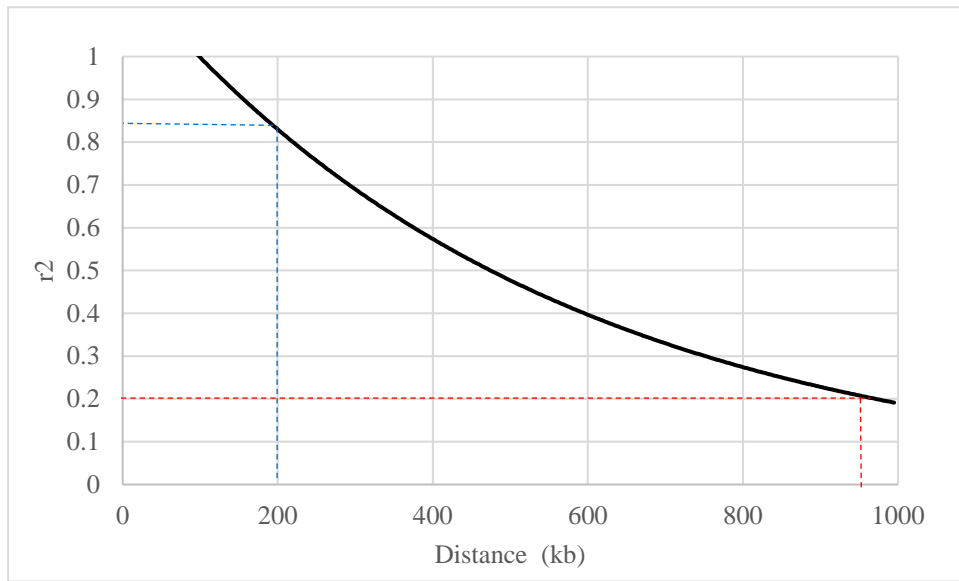

Figure S1 Linkage disequilibrium decay in the mapping population.

Supplement: Supplementary file 1 [file Image_1.pdf]
